# Nurse Level of Education, Quality of Care and Patient Safety in the Medical and Surgical Wards in Malaysian Private Hospitals: A Cross-Sectional Study

**DOI:** 10.5539/gjhs.v7n6p331

**Published:** 2015-04-23

**Authors:** Hamzah Abdul Rahman, Mu’taman Jarrar, Mohammad Sobri Don

**Affiliations:** 1 College of Business, Universiti Utara Malaysia, Kedah, Malaysia

**Keywords:** nurse level of education, teaching hospitals, hospital size, training, quality of care, patient safety, outcomes of care

## Abstract

**Background and Objective:**

Nursing knowledge and skills are required to sustain quality of care and patient safety. The number of nurses with Bachelor degrees in Malaysia is very limited. This study aims to predict the impact of nurse level of education on quality of care and patient safety in the medical and surgical wards in Malaysian private hospitals.

**Methodology:**

A cross-sectional survey by questionnaire was conducted. A total of 652 nurses working in the medical and surgical wards in 12 private hospitals participated in the study. Multistage stratified simple random sampling performed to invite nurses working in small size (less than 100 beds), medium size (100-199 beds) and large size (over than 200) hospitals to participate in the study. This allowed nurses from all shifts to participate in this study.

**Results:**

Nurses with higher education were not significantly associated with both quality of care and patient safety. However, a total 355 (60.9%) of respondents who participated in this study were working in teaching hospitals. Teaching hospitals offer training for all newly appointed staff. They also provide general orientation programs and training to outline the policies, procedures of the nurses’ roles and responsibilities. This made the variances between the Bachelor and Diploma nurses not significantly associated with the outcomes of care.

**Conclusions:**

Nursing educational level was not associated with the outcomes of care in Malaysian private hospitals. However, training programs and the general nursing orientation programs for nurses in Malaysia can help to upgrade the Diploma-level nurses. Training programs can increase their self confidence, knowledge, critical thinking ability and improve their interpersonal skills. So, it can be concluded that better education and training for a medical and surgical wards’ nurses is required for satisfying client expectations and sustaining the outcomes of patient care.

## 1. Introduction

Institute of Medicine (IOM) in their report To ‘Err Is Human’ reported that 98,000 deaths occurred annually in the United States of America as result of medical errors (IOM, 2000). In Australia, adverse events occurred for 17% of all admitted patients (Wilson et al., 1995). However, in Malaysia the demand and cost of care is increasing while lack of resources threaten sustainable performance of Malaysian health system (MOH, 2011a). Learning is essential to create positive and healthy work environments (Baumann, 2007), which can improve patient care outcomes (Aiken et al., 2012). Learning organizations are those which support professional development and knowledge sharing for long life learning (Baumann, 2007). Learning organizations are creating, acquiring and transferring knowledge (Wheelen, Hunger, Hoffman & Bamford, 2015), and convert it to improve their performance for solving functional problems (Yang, Watkins, & Marsick, 2004), which results in high service quality and staff satisfaction (Pantouvakis & Mpogiatzidis, 2013). Chen and Kuo (2011) show that there is a significant relationship between learning organizations and quality control; group learning affects quality control and improves the performance of hospitals. Many studies found that higher educated nurses are more associated with patient safety and quality of care (Aiken, Clarke, Cheung, Sloane & Silber, 2003; Cramer et al., 2011; Estabrooks et al., 2005; Tourangeau et al., 2006, 2007). For instance, Tourangeau et al. (2007) found that increasing the proportion of bachelorette prepared nurses by 10% led to decreased mortality by nine cases among one thousand discharged patients. Thus, hospital managers should prepare and attract better educated nurses which can better navigate today’s healthcare complexity to deliver best practices consistent with current evidence. However, nurses with Bachelor degree were limited in Malaysia (Yaakup, Eng, & Shah, 2014). According to the Malaysian Ministry of Health (MOH) Annual Report (2011) the performance of nurses working in private hospitals were lower than nurses working in public hospitals (MOH, 2011b). Thus, the main purpose of the study is to explore the effect of nurse level of education on quality of care and patient safety in Malaysian private hospitals.

## 2. Methodology

### 2.1 Design

A cross-sectional survey was conducted at the individual nurse level of analysis of nurses working in the medical and surgical wards in Malaysian private hospitals. Medical and surgical wards were chosen because they deliver multidisciplinary level of care such as medical cardiology, gastroenterology, urology, oncology, nephrology, orthopedics, and ear, nose and throat care (Coetzee, Klopper, Ellis & Aiken, 2013).

### 2.2 Sampling

Multistage stratified simple random sampling was performed to collect data from nurses working in all shifts in the medical and surgical wards. The stratified random sampling offers more homogeneity within the stratum and higher heterogeneity among group of strata which produce a “mirror image of the population” (Sekaran & Bougie, 2010). The simple random sampling of each stratum ensure each hospital and nurse have an equal chance to be chosen randomly (Sekaran & Bougie, 2010). The inclusion criteria of nurses are those nurses’ registered by the MOH Malaysia and delivering direct inpatient care in the medical and surgical wards. The inclusion criteria of hospitals are those registered in the Association of Private Hospitals of Malaysia. The number of beds used to evaluate hospital size according to the current nursing literature as follows (Gok & Sezen, 2013; K. H. Lee & S. B. Yang, 2009):

1) Small size: less than 100 beds.
2) Medium size: 100-199 beds.
3) Large size: over than 200 beds.

### 2.3 Operationalization and Measurement

The outcomes of care reflect the end result of the caring process (Harvey, 2004). The outcomes of care in the study consist of two dependent variables: quality of care and patient safety. Items used of measuring the study variables were internationally validated (Aiken et al., 2012; Coetzee et al., 2013; Van Bogaert, Clarke, Vermeyen, Meulemans & Van de Heyning, 2009). For measuring quality of care, nurses were asked to grade the overall quality of care in the last shift and during the last year (Van Bogaert, Meulemans, Clarke, Vermeyen & Van de Heyning, 2009). Furthermore, nurses were asked whether they will recommend the hospital to their friends and family if they need hospital care or as good place to work (Coetzee et al., 2013).

Patient safety is prevention of any potential harm for the hospitalized patients (Groene et al., 2010). According to the current nursing literature, the most common potential harm in the medical and surgical wards are nosocomial infection, pressure ulcer, patient fall, medication errors, readmission and patient and family complaints (Laschinger & Leiter, 2006; Van Bogaert et al., 2014; Weingart et al., 2011). Thus, nurses were asked to report their degree of agreement of the overall rating of the frequency of these events. Furthermore, nurses were asked to rate the overall patient safety in their unit using five point Likert scale, which is validated from the Agency for Healthcare Research and Quality survey of patient safety (Aiken et al., 2012; Coetzee et al., 2013; You et al., 2013). The nurse level of education was measured by asking the nurse to indicate his/her education and choosing among three categories; Bachelor, Diploma or others (Boumans, Landeweerd, & Visser, 2004).

Back-To-Back translation and pilot study are performed to make sure that questionnaire is reliable and free of mistakes, wrong wording or changes in the meaning. The coefficient of Cronbach’s alpha is indicated internally consistent and the adequate instrument used in the study. The Cronbach’s alpha coefficient of quality of care and patient safety was 0.75 and 0.85 respectively, which are above the recommended level of 0.70 (Pallant, 2011; Sekaran & Bougie, 2010). Multiple regression analysis performed using the SPSS software version 21.0 to investigate the impact of nurse level of education on quality of care and patient safety at p<0.05 level of significance.

## 3. Results

A total 652 nurses working in the medical and surgical wards with 61.8% response rate participated in the study from 12 private hospitals in Malaysia. The demographic characteristics of respondents indicated that 99.0% of nurses were Malaysian, and 97.6% of nurses were female. The respondents in the study were from the three ethnic groups: 60.0% Malay, 21.6% Chinese and 14.2% Indian. In term of employment status, 98.1% were full time while only 1.9% were part time nurses. A total 60.9% of nurses were working in teaching hospitals, while 39.1% from non-teaching hospitals. The teaching hospitals in the study refer to those hospitals awarding a degree of nursing. Additionally, 84.6% of respondents held a Diploma in nursing, while only 10.3% hold a Bachelor degree in nursing and 5.1% other (the others included a nurse who had an associate degree in nursing or higher education).

The nurse level of education construct include three categories, thus j – 1 dummy variables to capture all information for each category required to be compared with the reference group (Cohen, Cohen, West & Aiken, 2003; Hardy, 1993; West, Aiken & Krull, 1996). The reference group should be well defined in order to clearly interpret the study result. Furthermore, it should contain a sufficient number of respondents and should not be “others category” (Cohen et al., 2003; Hardy, 1993; West et al., 1996). Thus, the reference group was nurses with Diploma. Multiple regression analysis of the effect of nurse level of education on the outcomes of care explored in two regression models. The first model was explored the effect of nursing education on quality of care, whereas the second regression model was explored the effect of nursing education on patient safety.

### 3.1 Model 1: Nurse Level of Education and Quality of Care

Nurse level of education included two dummy variables. Table 1 shows the multiple regression analysis result of its impact on quality of care in order to test the hypothesis:

**Table 1:** Regression analysis result of nurse level of education on quality of care

Variables	Unstandardized Coefficients B	Std. Error	Standardized Coefficients β	t	Sig.
(Constant)	3.74	0.03		146.60	0.00

Diploma (R)					
Bachelor degree	-0.07	0.08	-0.04	-0.86	0.39
Other education	0.12	0.11	0.05	1.11	0.27

R2	0.004				
F value	1.07				
Significance of F value	0.34				

Significant level: ***: p<0.001; **: p<0.01; *: p<0.05. (R): Reference group.

The alternative hypothesis H1: nurse level of education is associated with quality of care.

The null hypothesis H1_0_: nurse level of education is not associated with quality of care.

The result of the regression analysis as shown in Table 1 revealed that F = 1.07 and P value = 0.34 indicated that the study failed to reject the null hypothesis H1_0_. So, the relationship of nurse level of education is not significantly affecting quality of care. The R^2^ indicates that nurse level of education variable predicted only 0.004 of variances in quality of care and not significant at level p<0.05. Moreover, nurses with Bachelor degree (B=-0.07, t=-0.86, p=0.39) are not significantly affecting quality of care at p<0.05 significance level compared with those have a Diploma. Thus, the hypothesis H1 is not supported.

### 3.2 Model 2: Nurse Level of Education and Patient Safety

Table 2 provided the result of multiple regression analysis of the nurse level of education on patient safety in order to test the hypothesis:

**Table 2:** Regression analysis result of nurse level of education on patient safety

Variables	Unstandardized Coefficients B	Std. Error	Standardized Coefficients β	t	Sig.
(Constant)	3.59	0.03		135.42	0.00

Diploma (R)					
Bachelor degree	-0.05	0.08	-0.03	-0.62	0.53
Other education	-0.03	0.11	-0.01	-0.26	0.80

R2	0.001				
F value	0.22				
Significance of F value	0.81				

Significant level: ***: p<0.001; **: p<0.01; *: p<0.05. (R): Reference group.

The alternative hypothesis H2: nurse level of education is associated with patient safety.

The result of the regression analysis as shown in Table 2 revealed that F = 0.22 and P value = 0.81 indicated that the study failed to reject the null hypothesis H2_0_. So, nurse level of education is not significantly affecting patient safety. The R^2^ indicates that nurse level of education variable predicts only 0.001 of variances in patient safety and not significant at level p<0.05. Moreover, nurses with bachelor degree (B=-0.05, t=-0.62, p=0. 53) is not significant affecting patient safety at p<0.05 significance level compared with those have a Diploma. Thus, the hypothesis H2 is not supported.

## 4. Discussion

Regression analysis of the impact of nurse level of education as reported in Table 1 and Table 2 indicated insignificant impact on both quality of care and patient safety at p<0.05 significance level. These findings are inconsistent with previous studies. Many studies found that nurses with higher education are significantly associated with delivering high quality of care and patient safety (Aiken et al., 2003; Cramer et al., 2011; Estabrooks et al., 2005; Tourangeau et al., 2007, 2006). For instance, Tourangeau et al (2007) found that increasing the proportion of bachelorette nurses by 10% significantly led to decrease in the mortality rate by nine cases among a thousand discharged patients (Tourangeau et al., 2007). Furthermore, highly educated and involved healthcare providers help to be more patient centered in order to improve quality of care and patient safety (Ferguson et al., 2007). However, respondents demographics discussed in section 3 revealed that 355 (60.9%) of respondents who participated in the study worked in teaching hospitals. In teaching hospitals, all new staff should given general orientation program and training to outline the policies, procedures and their role and responsibilities in order to improve quality of patient care (Scott, Poole, & Jayathissa, 2008). So, the conclusion is that Malaysian private hospitals have structured nursing orientation and training program of newly appointed staff, which made the variances of nurse level of education insignificantly associated with quality of care and patient safety. This was supported by a comparative study between the Malaysian and the Australian healthcare systems, where there were compulsory training programs for staff prior to entry into the general practice and a continuous professional development programs which are important for sustaining the outcomes of care (Khoo & Richard, 2002).

This point of view was supported by a number of studies in the Malaysian hospitals (Chiu, 2006; Ludin, Parker & Arbon, 2014). A study conducted in four Malaysian public hospitals supported this point of view (Ludin et al., 2014). Teaching hospitals had commitment to teaching and training of their staff (Ludin et al., 2014). Furthermore, in Malaysia there is a four weeks post registration training program conducted widely as a venture between an Australian university and Malaysian private hospitals (Chiu, 2005). The post registration training program for nurses in Malaysia help to upgrade the Diploma nurses (Chiu, 2006). Training program increased their self confidence, knowledge, critical thinking ability and their interpersonal skills (Chiu, 2006). So, it can be conclude that better education and training for a medical and surgical wards staff nurses is required for satisfying client expectations, sustaining the outcomes of care and improving quality and patient safety. Training and learning by providing skills for healthcare professionals is a key for optimizing quality and patient safety (Scott et al., 2008; Siriwardena, 2006; Valero et al., 2009).

## 5. Conclusion

There is no significant effect of the level of education of nurses working in the medical and surgical wards in Malaysian private hospitals because they have structured training programs of their appointed staff. This made the variances of nurse level of education insignificantly associated with quality of care and patient safety. Training and learning by providing skills for healthcare professionals is a key for optimizing quality and patient safety. Thus, hospital management should be prepared to educate nurses in order to optimize the outcomes of patient care. Furthermore, all new staff should be given a general orientation program and training to outline the policies, procedures and their role and responsibilities in order to improve quality of patient care. However, education and training alone is not effective to change practice. Thus, multi-level interventions are required in addition to education and training in order to sustain the outcomes of care. Further research is recommended to include staffing and the work environment factors to predict the outcomes of care.
